# Child malnutrition in the Democratic Republic of the Congo: A Bayesian Gaussian regression analysis using the 2023–24 demographic and health survey

**DOI:** 10.1371/journal.pone.0360056

**Published:** 2026-10-08

**Authors:** Zacharie Tsala Dimbuene, Hugues Sampasa-Kanyinga

**Affiliations:** 1 School of Population and Development Sciences, University of Kinshasa, Kinshasa, Democratic Republic of the Congo; 2 Faculty of Medicine, University of Kinshasa, Kinshasa, Democratic Republic of Congo; Jahangirnagar University, BANGLADESH

## Abstract

**Background:**

Child stunting remains a major public health challenge in the Democratic Republic of the Congo (DRC), reflecting persistent inequalities in child nutrition and health outcomes. This study examines the distribution and spatial variation of height-for-age z-scores (HAZ) among children under five, while also assessing the influence of selected demographic and socioeconomic factors.

**Methods:**

Data were obtained from the 2023–2024 Demographic and Health Survey (DHS-8). A structured additive regression model was then applied to assess the effects of child age and maternal education, allowing for nonlinear relationships through smooth functions. To capture geographic heterogeneity, spatial effects were decomposed into structured (spatially correlated) and unstructured (area-specific random) components, enabling the identification of regional clustering and residual province-level variation in stunting.

**Results:**

Geographic analysis reveals substantial regional disparities in stunting prevalence across the 26 provinces of the DRC. The province of Kwilu emerges as a major hotspot with very high levels of stunting, while several provinces fall within medium to high prevalence categories, indicating that stunting is a widespread issue across the country. Model results highlight important nonlinear relationships for certain predictors. Child’s age shows a strong negative trend with stunting risk: younger children have higher log-odds of stunting, and the risk decreases sharply with age. Maternal education displays a weaker and nonlinear association, with generally small effects across most education levels and greater uncertainty at higher levels of schooling. Spatial modeling further demonstrates that geographic factors significantly contribute to variation in stunting outcomes. Both structured spatial effects, indicating regional clustering, and unstructured spatial effects, reflecting province-specific deviations, contribute to the observed patterns. The combined spatial effect suggests persistent geographic disparities even after adjusting for covariates, although uncertainty varies across regions.

**Conclusions:**

The findings underscore the importance of geographic context and early childhood factors in shaping stunting outcomes in the DRC, highlighting the need for targeted, context-specific interventions to address persistent nutritional inequalities.

## Introduction

The UNICEF–WHO–World Bank Joint Child Malnutrition Estimates (JME) show that in 2024, an estimated 150.2 million children under five were stunted and 42.8 million were wasted globally, underscoring that the world is off track to meet the 2025/2030 global nutrition targets [1]. Height-for-age z-score (HAZ) is the primary indicator used to assess stunting, reflecting chronic undernutrition and long-term growth faltering [2,3]. According to WHO standards, stunting is defined as HAZ<−2 standard deviations from the median of the reference population [2]. This measure captures cumulative nutritional deficits and repeated infections during the first 1,000 days and beyond [4,5]. Stunting during early childhood is associated with impaired physical growth, delayed cognitive development, reduced educational attainment, and increased susceptibility to disease, all of which can have lifelong consequences for human capital formation and economic productivity [6,7]. Moreover, child malnutrition contributes substantially to under-five morbidity and mortality, making it a critical barrier to improving child health outcomes globally [5].

The burden of childhood stunting remains particularly high in the Democratic Republic of the Congo (DRC), a country characterized by persistent poverty, limited health infrastructure, political instability, and recurrent armed conflicts [8,9]. These structural challenges affect food security, healthcare access, and household living conditions, thereby increasing the vulnerability of young children to chronic undernutrition. Despite national efforts and international initiatives aimed at improving maternal and child health, stunting continues to affect a large proportion of children under five [10,11]. National averages, however, may conceal substantial heterogeneity in nutritional outcomes across regions and population groups [9]. Understanding the geographic distribution of child malnutrition is therefore essential for identifying vulnerable populations and designing effective public health interventions.

Previous studies investigating the determinants of child malnutrition in developing countries have primarily focused on individual, household, and socioeconomic factors such as maternal education, household wealth, sanitation, and child characteristics [8,12]. While these factors are critical determinants of nutritional status, they may not fully capture broader contextual influences related to geography and environment. Geographic location can act as a proxy for distal determinants such as food availability, agricultural conditions, health service accessibility, infrastructure, environmental exposure, and the consequences of conflict [9,13,14]. As a result, spatial context may shape child nutritional outcomes beyond what can be explained by individual or household characteristics alone. Recognizing the importance of spatial context, recent methodological advances in spatial statistics allow researchers to explore geographic patterns of health outcomes more comprehensively. Geo-additive semi-parametric models provide a flexible framework to simultaneously account for nonlinear effects of continuous covariates and spatial dependence across neighboring regions [15–20]. Such approaches enable researchers to disentangle structured spatial variation—reflecting regional clustering—from unstructured area-specific heterogeneity, thereby offering deeper insight into the geographic drivers of health inequalities.

This study extends the spatial analytical framework proposed in previous research by examining the distribution and spatial determinants of stunting among children under five in the DRC [9].

Using height-for-age z-scores and spatial modeling techniques, the paper investigates how demographic factors, maternal characteristics, and geographic location contribute to variations in child nutritional outcomes. By identifying persistent spatial disparities after accounting for key covariates, the study aims to provide evidence that can inform geographically targeted nutrition and health policies in the country. Ultimately, a better understanding of the spatial structure of childhood malnutrition can support more efficient allocation of resources and help policymakers design interventions that address both immediate and underlying determinants of stunting across regions of the DRC.

## Methods

### DRC context: DHS 2023–2024

The DRC is a Central African country divided into 26 provinces, including the capital city-province of Kinshasa (Fig 1). It is the second-largest country in Africa and the eleventh-largest globally by land area. With an estimated population of approximately 112 million, it is the fourth-most populous country in Africa.

**Fig 1:**
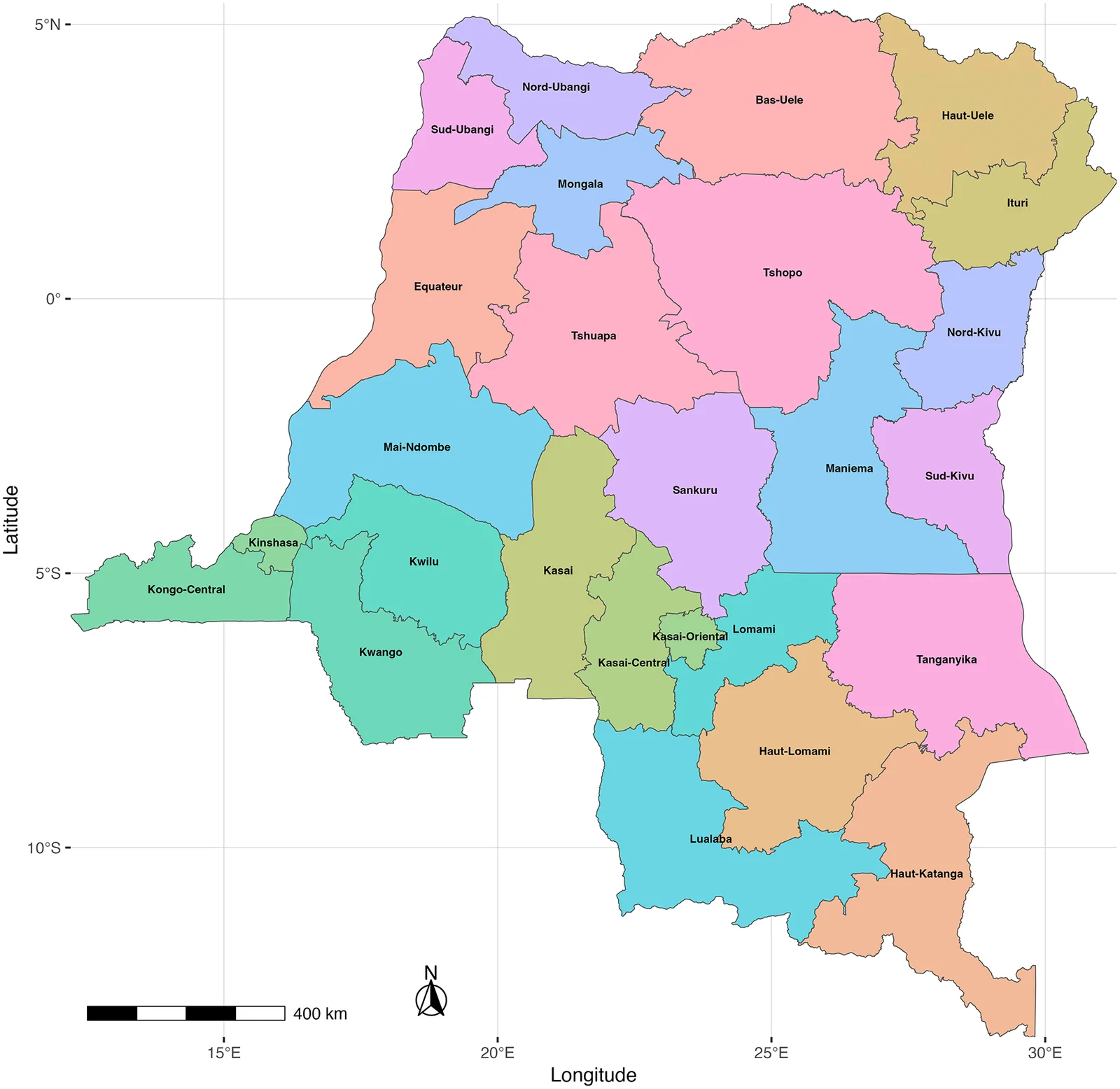
Map of 26 provinces of Democratic Republic of the Congo. Notes: Maps generated using the R package rgeoboundaries, which accesses the GeoBoundaries database of political administrative boundaries (CC BY 4.0, https://www.geoboundaries.org).

The DRC recently completed its Demographic and Health Survey (DHS-8; EDS-RDC III), with fieldwork conducted between October 2023 and January 2024. The survey is nationally representative and provides rich information on child anthropometry, water, sanitation and hygiene (WASH) conditions, household assets, morbidity, infant and young child feeding (IYCF) practices, and georeferenced cluster data. These features enable detailed subnational and urban–rural analyses [21].

Complementary situational assessments reinforce the survey’s relevance. Acute malnutrition analyses from the Integrated Food Security Phase Classification (IPC) and humanitarian needs overviews from OCHA highlight elevated wasting in specific health zones, alongside persistent food insecurity driven by conflict, displacement, and disruptions to basic services [22,23]. The Global Nutrition Report further underscores chronic vulnerabilities linked to poverty, inadequate infrastructure, and prolonged instability [24]. Together, these sources point to a nutrition landscape shaped not only by household-level determinants but also by broader environmental and systemic constraints.

### Variable measurement

#### Outcome Variable: Height-for-Age (HAZ).

Most empirical studies define stunting using the WHO threshold of a height-for-age z-score (HAZ) below –2, operationalized as a binary outcome rather than a continuous measure. While this dichotomous indicator facilitates comparability across studies, it compresses substantial variation in child growth outcomes. Treating HAZ as binary discards information about the severity of deviation from the reference standard—for instance, a child with HAZ = –2.01 is classified identically to one with HAZ = –4.5, yet both are considered fundamentally different from a child with HAZ = –1.99, despite nearly identical nutritional status. This loss of granularity reduces statistical power, obscures dose–response relationships, and masks gradients in chronic growth faltering. In this study, we therefore model height-for-age as a **continuous outcome**, preserving the full distribution of growth trajectories and enabling the detection of nonlinearities and heterogeneous effects.

#### Key Exposures.

The analysis focuses on two primary exposures widely documented in the literature. First, child’s age was found to be the strongest predictor of child growth [25]. Therefore, understanding the relationship between child’s age and linear growth is of paramount importance. Second, maternal education was significantly associated with child’s linear growth in previous studies [26,27].

In addition, longitudinal studies have analyzed the joint effects of child’s age and maternal education. For more details, see [28]. While many studies categorize these variables, we exploit the granularity of DHS data by modeling them as **continuous variables**. This approach allows for the identification of **nonlinear relationships** with HAZ.

#### Covariates and Confounders.

We adjust for a comprehensive set of covariates identified in prior literature, spanning multiple levels: (*i*) **Child-level:** sex; (*ii*) **Mother-level:** age; (*iii*) **Household-level**: household wealth index; and (*iv*) **Context-level:** urban/rural residence, province.

### Analytical strategy

#### Model Formulation.

Let $Y_{i}$ denote the continuous height-for-age z-score (*HAZ*) for child i, located at spatial coordinates $s_{i}$ and residing in province $p_{i}$.

We specify a Bayesian spatially varying coefficient (SVC) model:


$$\begin{array}{c} Y_{i}\sim N\left(\mu_{i},\sigma^{\text{2}}\right) \end{array}$$

with linear predictor:


$$\begin{array}{c} \mu_{i}=\beta_{\text{0}}+\beta_{\text{1}}{\text{Age}}_{i}+\beta_{\text{2}}{\text{Sex}}_{i}+\beta_{\text{3}}{\text{Wealth}}_{i}+u_{p_{i}}+f_{\text{spatial}}\left(s_{i}\right)+f_{\text{spatial},\text{ edu}}\left(s_{i}\right)\cdot {\text{Edu}}_{i} \end{array}$$

where: $\beta_{\text{0}},\beta_{\text{1}},\beta_{\text{2}},\beta_{\text{3}}$ are fixed effects; $u_{p_{i}}\sim N\left(\text{0},\sigma_{u}^{\text{2}}\right)$ is a province-level random effect; $f_{\text{spatial}}\left(s_{i}\right)$ is a spatial intercept; $f_{\text{spatial},\text{ edu}}\left(s_{i}\right)$ is a spatially varying coefficient for maternal education. In the equation, $\sigma^{\text{2}}$ is the residual variance of $Y_{i}$ conditional on $\mu_{i}$ (i.e., the unexplained variability remaining after accounting for the fixed effects, the province random effect, and the spatial terms).

#### Spatial modeling of child malnutrition.

The spatial process is modeled using a Gaussian Random Field (GRF), approximated via a finite basis representation over a triangular mesh:


$$\begin{array}{c} f_{\text{spatial}}(s)\approx \sum_{k=\text{1}}^{K}\phi_{k}(s)w_{k} \end{array}$$

where:

- $\phi_{k}(s)$ are piecewise linear basis functions
- $w_{k}\sim N\left(0,Q^{-1}\right)$ are Gaussian weights
- Q is a precision matrix derived from a Matérn covariance function

Similarly, the spatially varying slope is defined as:


$$\begin{array}{c} f_{\text{spatial},\text{ edu}}(s)\approx \sum_{k=\text{1}}^{K}\phi_{k}(s)w_{k}^{\text{edu}} \end{array}$$

#### Bayesian inference.

Bayesian inference is conducted using the **BayesX** framework, which implements full posterior estimation via Markov Chain Monte Carlo (MCMC). The estimation strategy includes:

- Gibbs and Metropolis updates for Gaussian model components
- Iteratively Weighted Least Squares (IWLS) proposals for non-Gaussian settings
- Gamma hyperpriors for smoothing parameters and variance components

Posterior summaries include credible intervals and spatial effect maps [29,30]. The **R2BayesX** interface enables implementation within R using the bayesx() function. It supports multiple estimation strategies, including:

- MCMC estimation
- Restricted Maximum Likelihood (REML)
- STEP-based penalized estimation with variable selection

Structured additive components—such as nonlinear effects, spatial Markov random fields, and random effects—are specified using the sx() term constructor [29,31,32].

### Ethical approval and consent to participate

An online request was submitted to the DHS programme’s official website (https://www.dhsprogram.com/data) for this specific study and ethical clearance and permission to use the datasets were granted. Confidentiality was maintained throughout, and informed consent was obtained from all respondents during the original data collection.

## Results

### Descriptive results

Table 1 below shows sample characteristics which consisted of 10,745 children aged 0–59 months. Overall, the average *HAZ* was −1.823 (SD = 1.750). Further analyses disaggregated HAZ by child’s gender and urban residence (Fig 2); and province of residence (Fig 3). Findings indicated that HAZ does not differ by urban residence while it differs significantly between girls and boys (Table 1 and Fig 2).

**Table 1:** Descriptive characteristics of the sample of children under five years, Democratic Republic of the Congo (DRC DHS 2023–2024).

Variable	Mean (SD)	N (%)
***Outcome: Height-for-age z-score (HAZ)***		
Entire Sample	−1.823 (1.750)	
Females^+^	−1.731 (1.734)	
Males	−1.912 (1.761)	
Rural children^++^	−1.816 (0.021)	
Urban children	−1.839 (0.029)	
***Key exposures***		
Child age (months)	28.075 (17.642)	
Mother’s education (years)	6.500 (4.245)	
***Covariates and Confounders***		
**Child sex**		
Male		5,473 (50.9%)
Female		5,272 (49.1%)
**Mother employment**		
Is not working		4,800 (44.7%)
Is working		5,945 (55.3%)
**Place of residence**		
Rural		7,130 (66.4%)
Urban		3,615 (33.6%)
**Total (*N*)**		**10,745**

+ The difference between females and males was statistically significant. On average, HAZ was −1.912 for males and −1.731 for females (A difference of −0.181; *p* < 0.001).

++ The difference between urban and rural children was not statistically significant.

**Fig 2:**
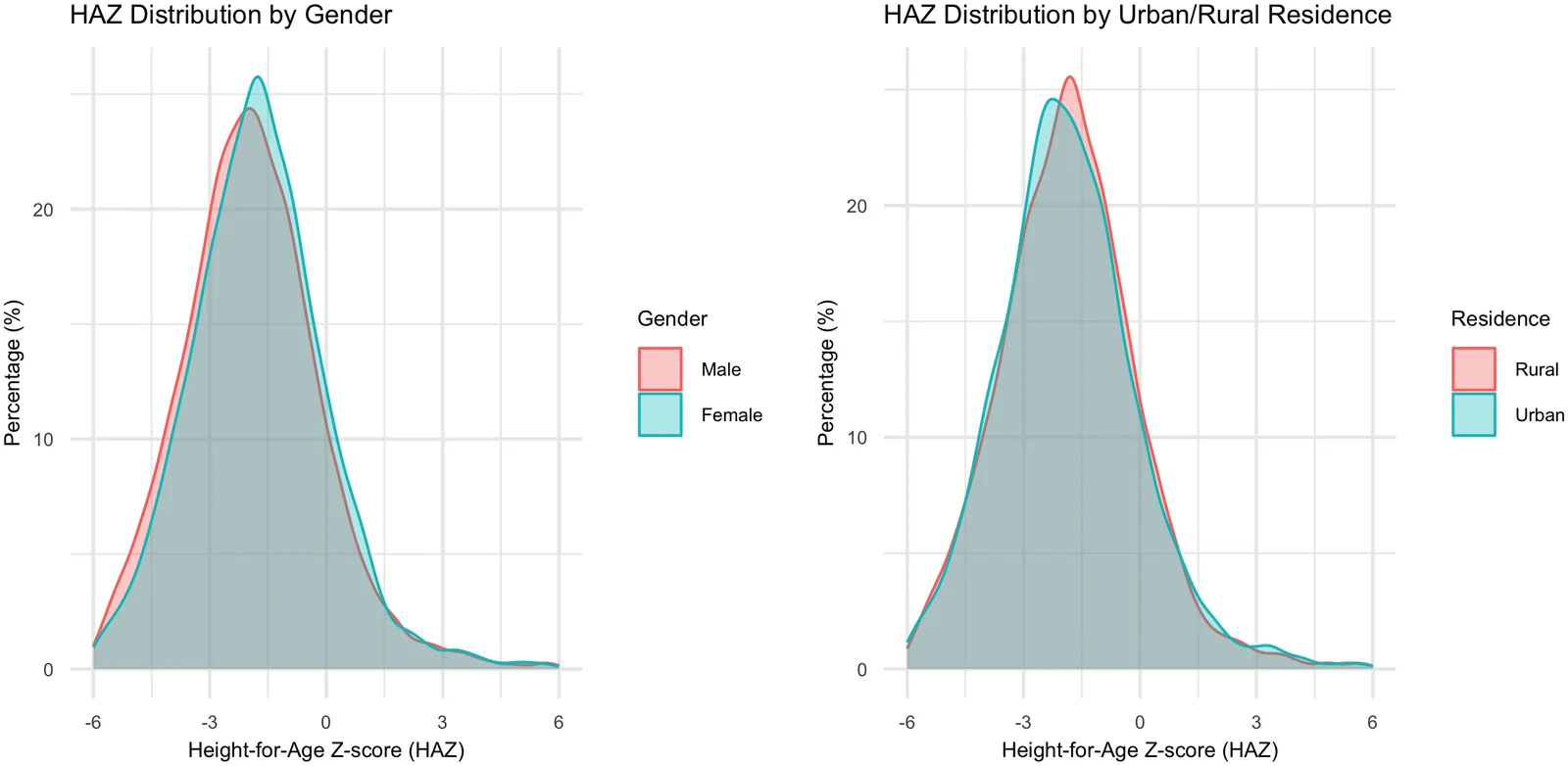
Distributions of Height-for-Age Z-scores (HAZ) by child’s gender and place of residence.

**Fig 3:**
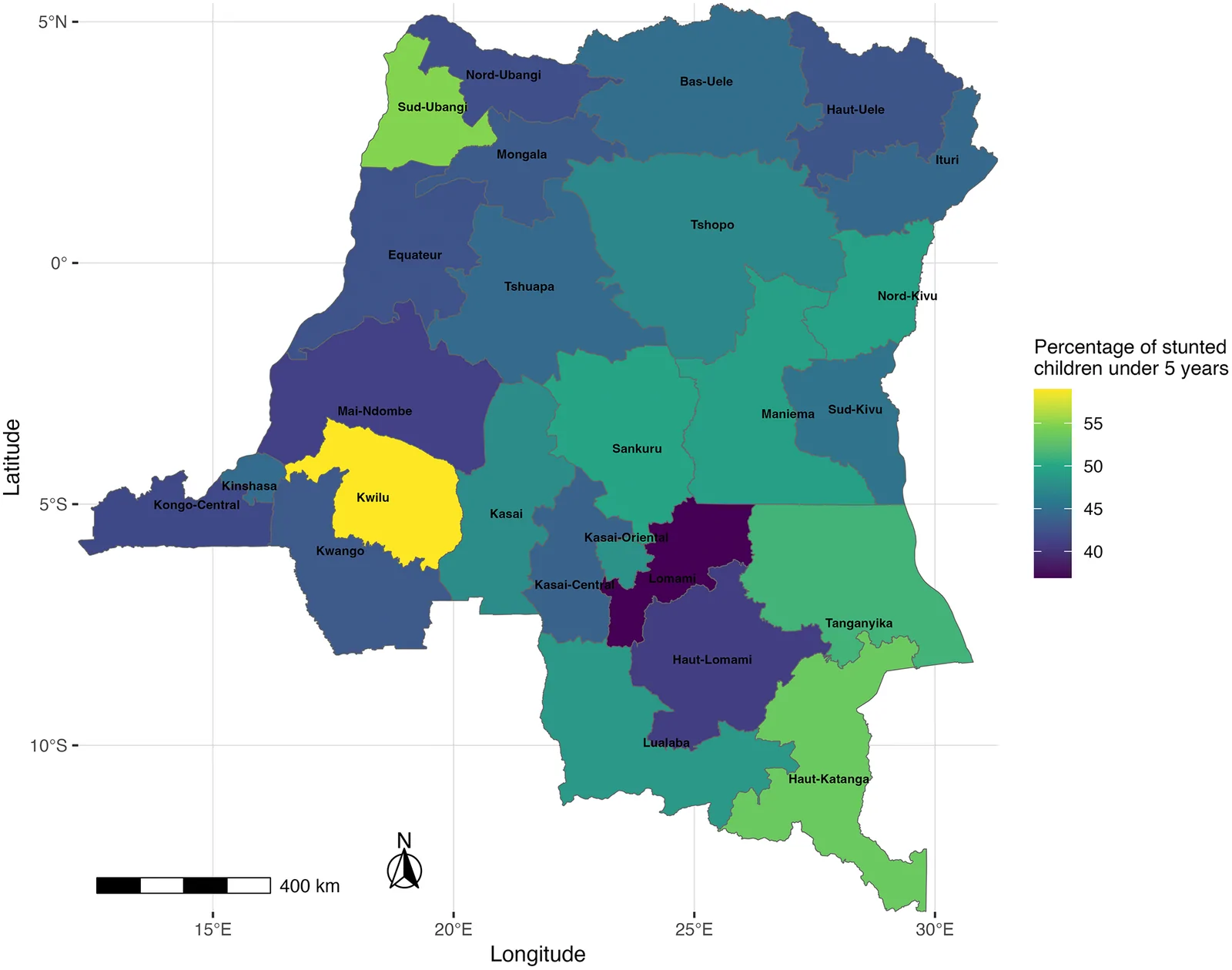
Prevalence of stunted children (<−2 HAZ) in Democratic Republic of Congo. Notes: Maps generated using the R package rgeoboundaries, which accesses the GeoBoundaries database of political administrative boundaries (CC BY 4.0, https://www.geoboundaries.org).

Fig 2 displays the distribution of HAZ by gender and place of residence. The distribution of Height-for-Age Z-scores (HAZ) revealed consistent patterns across subgroups. When stratified by gender, both boys and girls showed similar curves peaking around –2, though the male distribution was slightly more dispersed, suggesting marginally higher variability in growth outcomes. A comparable pattern emerged when comparing urban residence. With respect to residence, the urban curve is shifted slightly to the left of the rural curve, indicating marginally higher levels of stunting among urban children compared with rural children. This pattern is consistent with the lower mean HAZ observed among urban children relative to rural children in Table 1. These findings indicate that stunting remains widespread across demographic groups, with only subtle differences by sex or place of residence, underscoring the pervasive nature of child growth challenges in the study population.

Fig 3 and 4 shows initial insights of the spatial distribution of child malnutrition in the DRC.

**Fig 4:**
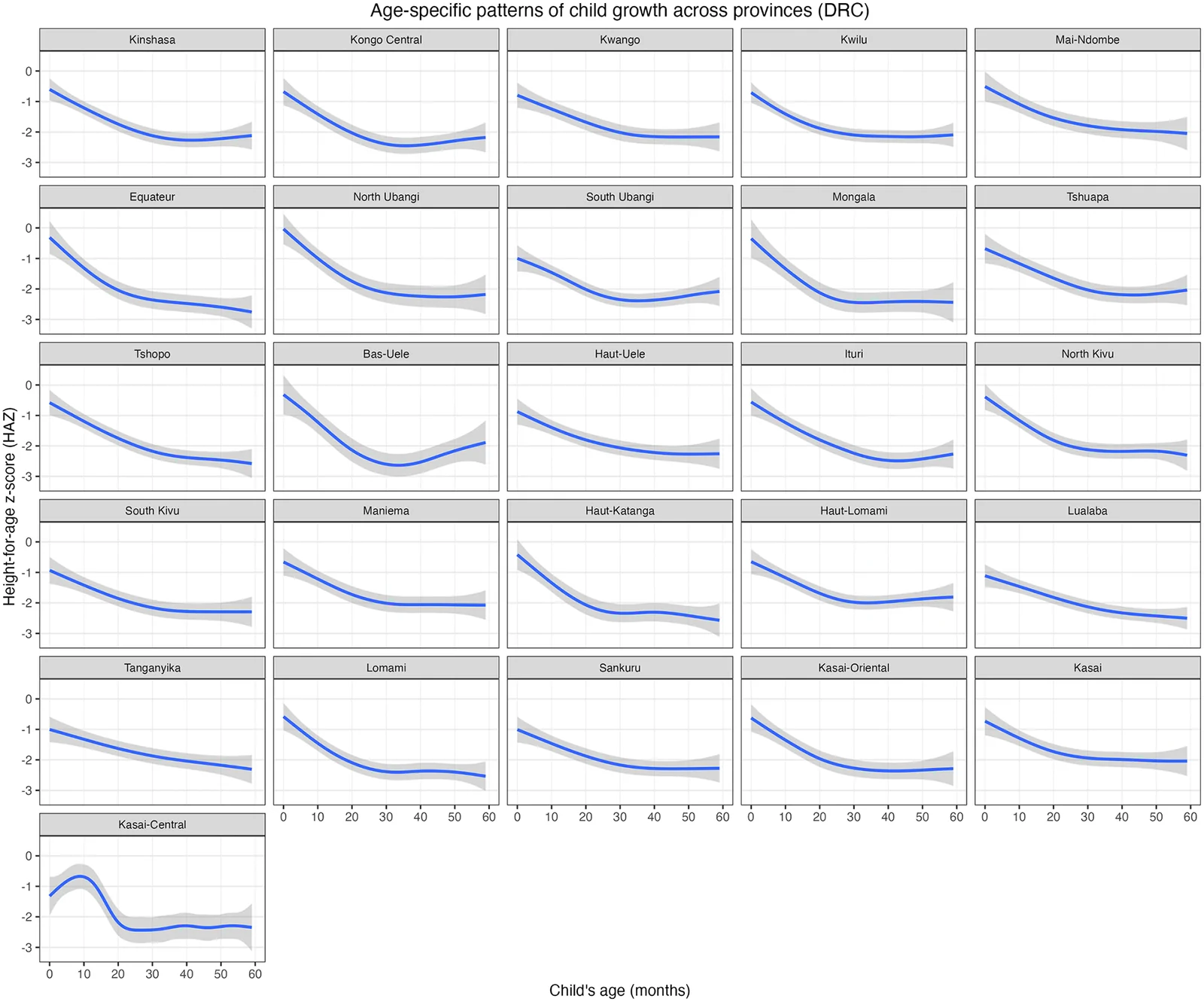
Nonlinear association between child age (in months) and height-for-age z-scores (HAZ) across provinces in the Democratic Republic of the Congo. Smoothed curves are estimated using generalized additive models (GAM), with shaded areas representing 95% confidence intervals.

The prevalence of stunting among children under five varies considerably across the 26 provinces of the DRC (Fig. 3). Clear spatial disparities emerge, with rates unevenly distributed and reflecting substantial regional inequality in child nutritional outcomes. The province of Kwilu stands out as a critical hotspot, recording very high prevalence and signaling urgent need for targeted nutritional and health interventions. Many provinces fall into the medium or high categories, underscoring that stunting remains a widespread public health concern beyond the highest-burden areas. In contrast, some central provinces such as Lomami and Kasai-Central are classified as very low, while provinces including Kongo-Central and Kwango fall into the low category. Together, these patterns highlight both the persistence and heterogeneity of chronic growth faltering across the country.

Fig 4 highlights age-specific patterns of child growth in the Democratic Republic of Congo. Clearly, the child growth in the country presents different patterns. While most provinces show linear child growth, others have U-shape (e.g., Bas-Uele, Kongo Central, South Ubangi). The province of Kasai-Central has even a very specific shape compared with other provinces. It has a bell-shaped before 24 months, and a flat shape after 24 months.

Fig 5 shows the smooth (nonlinear) effects of child age and maternal education (in years) on the outcome, expressed on the log-odds scale. Age is standardized; therefore, values below zero represent younger children, while values above zero represent older children. At lower (younger) ages, the effect is strongly positive, indicating higher log-odds of stunting among younger children. As age increases, the effect declines sharply, suggesting that the risk of stunting decreases with age. The confidence intervals are relatively narrow across most age values, indicating that the estimated age effect is precise. Maternal education appears to have a weak and nonlinear association with the outcome. Across most of the education range, the effect remains small and close to zero.

**Fig 5:**
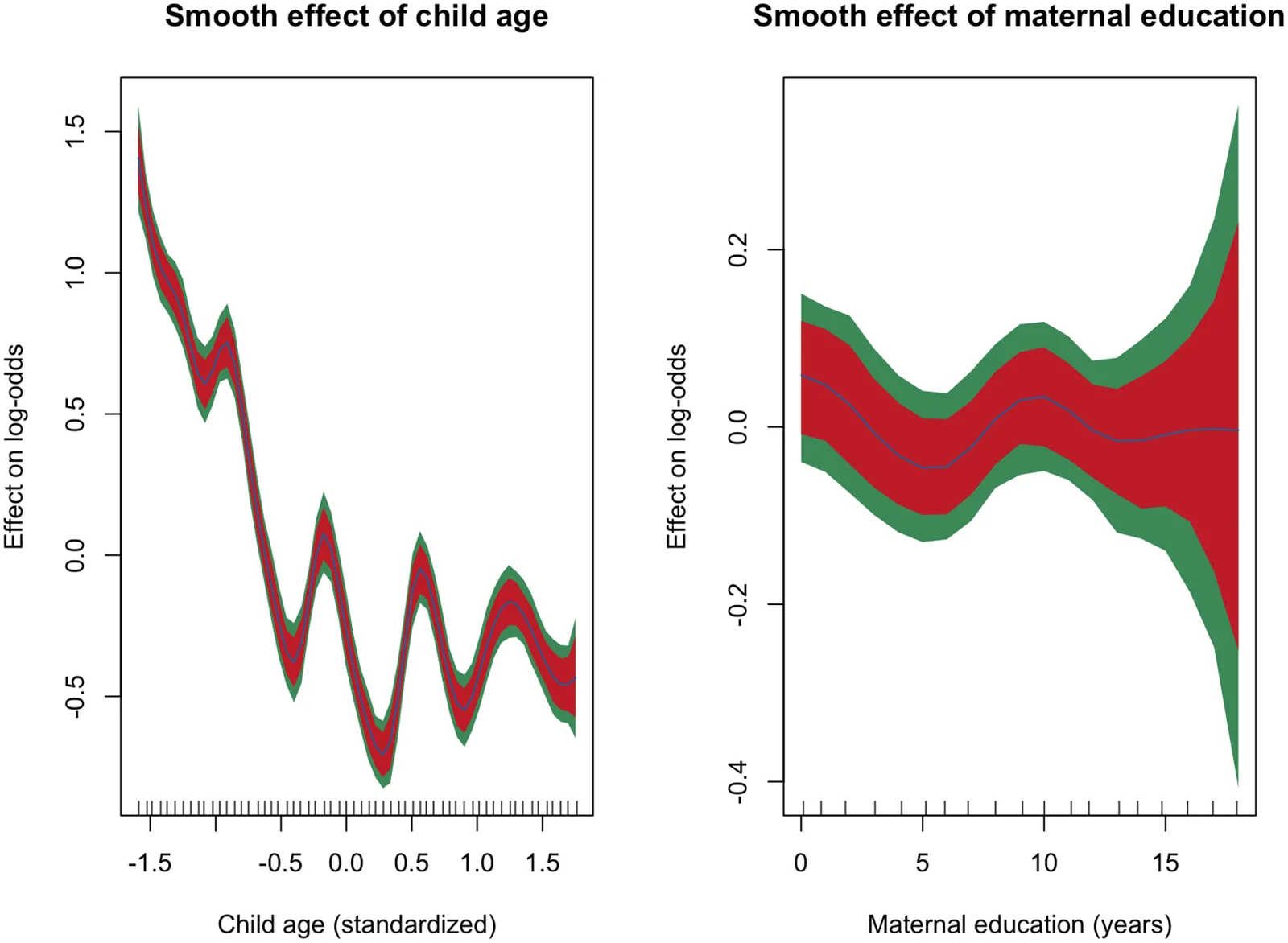
Nonlinear associations of child age and maternal education with height-for-age z-scores (HAZ) in Democratic Republic of Congo.

However, uncertainty increases at higher education levels, as reflected by the wider confidence intervals, which may indicate fewer observations in this range or reduced precision of the estimates. Overall, these findings suggest that the smooth term for maternal education is not strongly influential, except possibly within certain mid-range values.

Fig 6 presents four maps illustrating how spatial variation in the outcome is decomposed into structured and unstructured spatial effects, as well as the total spatial effect, along with the uncertainty associated with these estimates. Overall, the model suggests that geography plays an important role, with both regional clustering and area-specific variation contributing to differences in the outcome. The structured spatial effect provides evidence of spatial clustering, as neighboring regions display similar colors. In contrast, the unstructured spatial effect reflects area-specific random deviations that are not spatially correlated. The total spatial effect, which represents the sum of the structured and unstructured components, shows the overall residual spatial pattern after adjusting for covariates and highlights persistent geographic disparities. The uncertainty map indicates that precision varies across regions; therefore, some apparent extremes in the spatial effects may not be statistically robust. Additional details are provided in Table 2.

**Table 2:** Posterior estimates of fixed, nonlinear, and spatial effects on height-for-age z-scores (HAZ) among children under five in the Democratic Republic of the Congo.

Bayesian estimates	Mean	SD	2.5%	50%	97.5%
(Intercept)	−1.950	0.047	−2.044	−1.948	−1.863
Female child (vs male)	0.187	0.031	0.129	0.185	0.252
Mother employed (vs not employed)	−0.024	0.033	−0.090	−0.024	0.038
Urban residence (vs rural)	−0.037	0.040	−0.114	−0.037	0.040
Child age (nonlinear effect)	0.944	0.944	0.580	0.273	0.788
Maternal education (nonlinear effect)	0.004	0.004	0.004	0.001	0.003
Province-level spatial effect (MRF)	0.013	0.013	0.014	0.001	0.009

**Fig 6:**
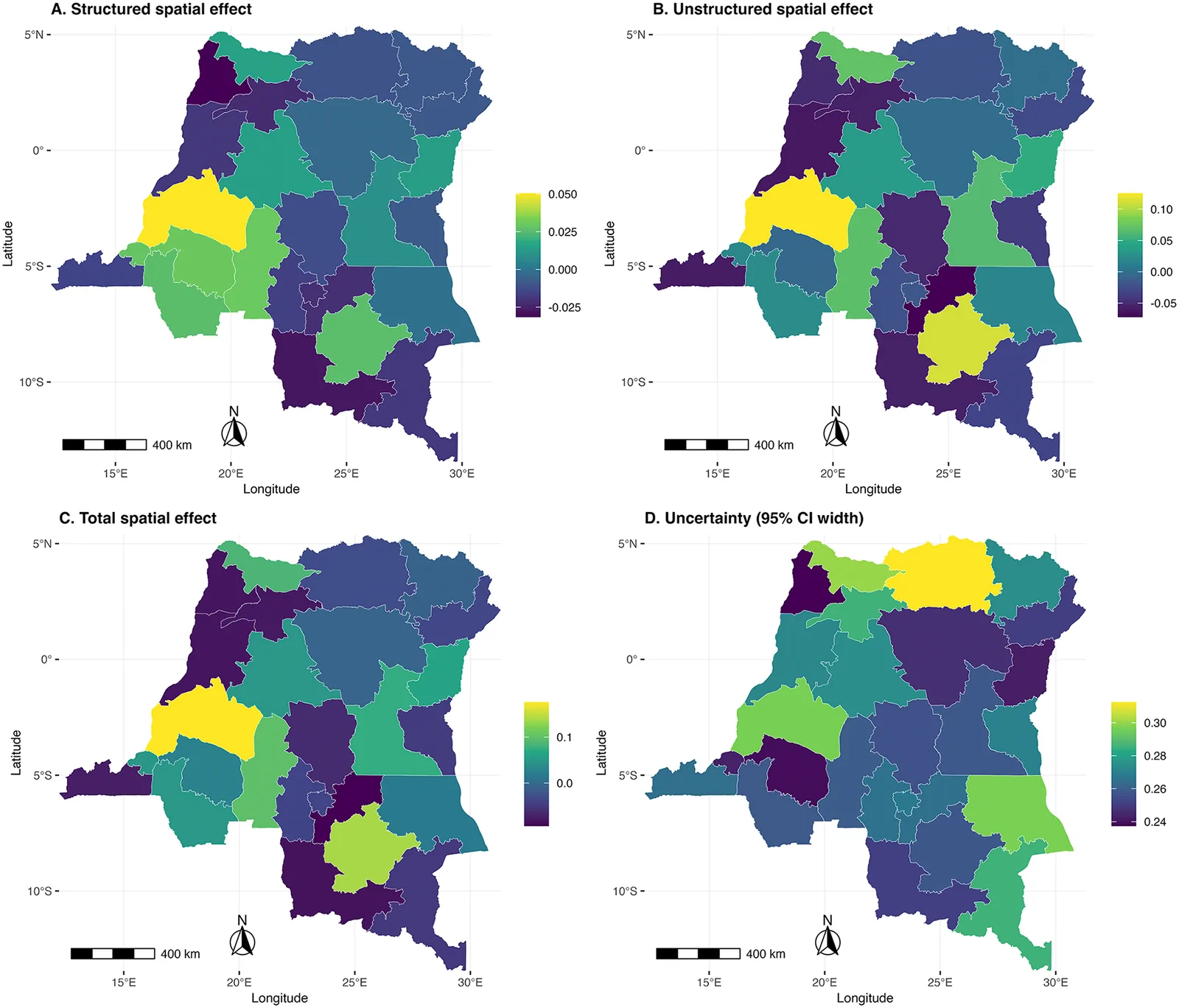
Structured, unstructured, and total spatial effects and uncertainty of child stunting in Democratic Republic of Congo. Notes: Maps generated using the R package rgeoboundaries, which accesses the GeoBoundaries database of political administrative boundaries (CC BY 4.0, https://www.geoboundaries.org).

Table 2 presents the posterior estimates of the Bayesian geoadditive model examining factors associated with height-for-age z-scores (HAZ) among children under five years in the Democratic Republic of the Congo. The estimated intercept was −1.95 (95% credible interval [CrI]: −2.04, −1.86), indicating generally poor linear growth outcomes in the study population. Female children had significantly higher HAZ than male children (posterior mean = 0.19; 95% CrI: 0.13, 0.25). In contrast, maternal employment status and urban residence were not significantly associated with HAZ, as their credible intervals included zero.

The nonlinear effects suggested that child age was an important determinant of HAZ, indicating substantial variation in growth patterns across age groups. Maternal education also exhibited a nonlinear association with HAZ, although the overall effect was relatively small. Furthermore, the province-level spatial effect demonstrated residual geographic variation in child nutritional status after controlling for observed covariates, suggesting the presence of unmeasured contextual factors influencing child growth across provinces.

## Discussion

This study examined the distribution and spatial determinants of height-for-age among children under five in the DRC using recent DHS data and Bayesian spatial modeling techniques. Several important findings emerge from the analysis. First, the distribution of HAZ indicates that children in the DRC remain, on average, below the WHO growth standard, reflecting the persistent burden of chronic undernutrition in the country. Second, substantial geographic disparities in stunting prevalence were observed across provinces. Third, nonlinear relationships were identified for child age and maternal education. Finally, the spatial model revealed residual geographic clustering even after adjusting for key socioeconomic and demographic covariates.

The results show that the distribution of HAZ scores is approximately normal and centered below the WHO reference median. This pattern suggests that growth faltering affects a broad proportion of children rather than being concentrated only among the most severely deprived households. Previous global analyses have similarly emphasized that stunting often reflects widespread structural deprivation rather than isolated cases of severe nutritional deficiency [3,5]. The small differences observed between boys and girls are also consistent with prior studies reporting minimal gender disparities in linear growth during early childhood in many low- and middle-income countries [4].

Clear spatial variation in stunting prevalence was evident across provinces, with some regions displaying substantially higher levels of chronic undernutrition. These geographic disparities likely reflect differences in poverty, food security, healthcare access, infrastructure, and exposure to conflict or displacement. In the DRC, protracted instability and disruptions to food systems and health services continue to affect child nutrition outcomes in several regions [22,24]. Similar spatial inequalities in child growth have been reported in other African settings, emphasizing the importance of geographic and contextual determinants [9,13]. Importantly, the spatial analysis revealed residual geographic clustering even after adjusting for demographic and socioeconomic variables [33]. Similar results were observed in Mozambique [34] and Ethiopia [35]. This finding suggests that unobserved contextual factors, such as agricultural conditions, environmental factors, or local health service availability may contribute to regional differences in child growth outcomes. These results highlight the value of spatial modeling approaches in identifying areas where additional structural or environmental factors may influence nutritional outcomes.

The nonlinear effect of child age observed in this study is consistent with evidence that growth faltering occurs primarily during the first two years of life. This period coincides with the transition to complementary feeding and increased exposure to infections, making it a critical window for nutritional interventions [4,12].

From a policy perspective, the findings emphasize the need for geographically targeted nutrition strategies in the DRC. National averages may obscure significant regional inequalities, potentially limiting the effectiveness of uniform national interventions. Strengthening maternal and child health services, improving food security, and investing in water and sanitation infrastructure, particularly in high-burden regions, could help reduce chronic undernutrition. Such interventions are especially important during the first 1,000 days of life, when improvements in nutrition yield the greatest long-term benefits for child development and human capital formation [4–6,36,37].

### Strengths and limitations

This study has several strengths. The use of recent nationally representative DHS data ensures that the findings reflect current nutritional conditions in the country. In addition, the application of Bayesian spatial modeling techniques allows for flexible modeling of nonlinear covariate effects while simultaneously capturing spatial dependence across regions. By using HAZ as a continuous outcome rather than a binary indicator of stunting, the analysis preserves more information about the distribution of child growth and enhances statistical power.

Nevertheless, several limitations should be acknowledged. First, the cross-sectional design of DHS data limits the ability to infer causal relationships between exposures and child growth outcomes. Second, some potentially important determinants—such as dietary diversity, food prices, agricultural productivity, or environmental conditions—may not be fully captured in the dataset. Third, DHS cluster coordinates are spatially displaced for confidentiality purposes, which may introduce uncertainty in fine-scale spatial analyses. Future research could benefit from integrating DHS data with environmental, climatic, or agricultural datasets to better understand the broader determinants of spatial variation in child nutrition.

## Conclusion

The study highlights the persistent burden and spatial heterogeneity of childhood stunting in the Democratic Republic of the Congo. The findings demonstrate that geographic context plays a significant role in shaping child nutritional outcomes beyond individual and household characteristics. Spatial analytical approaches therefore provide valuable tools for identifying high-risk regions and informing targeted public health strategies. Addressing chronic undernutrition in the DRC will require coordinated efforts that tackle both immediate nutritional needs and the broader structural determinants of child health.
