# Supplementary material for: Child malnutrition in the Democratic Republic of the Congo: A Bayesian Gaussian regression analysis using the 2023–24 demographic and health survey
Source: PLoS One. 2026 Oct 8;21(10):e0360056. doi: 10.1371/journal.pone.0360056 (PMC13648973; doi:10.1371/journal.pone.0360056)
Supplement: Supplementary file 1 [file pone.0360056.s001.docx]

**Point-by-point responses to Editor’s comments and Reviewers**

**Manuscript: PONE-D-26-15920**

**Child Malnutrition in the Democratic Republic of the Congo: A Bayesian Gaussian Regression Analysis Using the 2023–24 Demographic and Health Survey**

**Editor’s comments**

- In Fig 1, it is suggested that the scale, latitude, longitude, and north indication be added. Also, remove the title used in the map.
- In Fig 3, it is suggested that the scale, latitude, longitude, and north indication be added. Also, remove the title used in the map.
- In Fig 6, it is suggested that the scale, latitude, longitude, and north indication be added.
- Add strengths and limitations as a separate section.
- It is recommended to provide the reproducible code as a supplementary file.

**Responses to Editor’s comments**

Dear Editor,

The authors truly appreciate the opportunity to revise and resubmit the paper to Plos ONE. Please that all your comments/suggestions have been implemented in the manuscript: Scale, latitude, longitude and North arrow have been added in Figures 1, 3, and 6. As well, a supplementary file has been provided for reproducibility. Finally, “Strengths and limitations” as separate section was added in the manuscript.

**Review Comments to the Author**

Please use the space provided to explain your answers to the questions above. You may also include additional comments for the author, including concerns about dual publication, research ethics, or publication ethics. (Please upload your review as an attachment if it exceeds 20,000 characters)

**Reviewer #1: Manuscript Review report**

Manuscript title: Child Malnutrition in the Democratic Republic of the Congo: A Bayesian Gaussian Regression Analysis Using the 2023–24 Demographic and Health Survey
1. Summary of the research and overall impression
Research question, claims, conclusions and context: The authors explored the geographical distribution of the prevalence, and correlates for child malnutrition in the Democratic Republic of the Congo (DRC) based on the Height for age (HAZ) indicator and by fitting nonlinear Bayesian-Gaussian secondary regression analysis to data from DRC’s 2023–24 Demographic and Health Survey. They claim that the findings confirm the widespread prevalence of stunting in this vast country with important geographic inequalities. Thus, they conclude that their approach helps providing evidence for focusing interventions on most affected regions of the country and towards significant associated factors. This research reproduces the methods used elsewhere in Africa and other regions of the world confronted with child malnutrition, and provide similar conclusions.
Strengths and weaknesses: The manuscript has the merit of using data from the Demographic and Health Survey 2023-2024 which is both a recent and a methodologically sound study covering the whole RDC. The analytical tools used permit to explore nonlinear associations using regression to control for potential confounders. The geographic mapping of stunting is very useful for focused interventions.
However, as the authors point out, the cross-sectional nature of the data does not permit making causal inferences about the factors contributing to child stunting. Moreover, the analysis is only powered to the level of the province which represents the intermediate level in the health structure of the DRC. The true operational level, much more suited for interventions, is the health zone (health district) level. In addition, in relation to child chronic malnutrition, the distinction made in this paper among the key covariates and the potential confounders is unclear.
Overall recommendation: The authors recommend to use spatial analytical tools to identify malnutrition high-risk regions and to inform targeted public health interventions. I agree with authors’ recommendation to use coordinated efforts to tackle both immediate needs and broader determinants of child health. I recommend that the authors take into account the minor issues raised below so that the review process of this manuscript proceed forward.

**Response: The authors agree with the thorough review above from Reviewer #1. The reviewer raised the concern that the “true operational level, much more suited for interventions, is the health zone (HZ)”. Although the authors do agree with the suggestion, the reviewer should also note that this is a “secondary data analysis” of Demographic and Health Survey. Therefore, it is hard to get reliable estimates at HZ level since DHS were not designed to obtain estimates at a much more granular level such as HZ. Many published papers using DHS datasets overlook the spatial distribution of their outcomes of interest. In this paper, we paid special attention on “spatial distribution” of child malnutrition in the DRC which, even though, it does not provide the full picture of the problem at lower administrative level (e.g., HZ) can be used as the benchmark to further address inequalities in the countries. For instance, findings showed that the province of Kwilu is the most affected in the country even though many other provinces are also affected. Researchers and policymakers might then ask what is happening in this province and what could be done.**

2. Evidence and examples
Major Issues
None

Minor Issues
a. Lines 1-3: Title: the authors should consider reformulating the title in order to clearly announce the content of the manuscript.
Example: Distribution of Child Malnutrition in the Democratic Republic of the Congo: A Bayesian Gaussian Regression Analysis Using the 2023–24 Demographic and Health Survey data.

**Response: The authors agree with Reviewer #1; however, the title of the manuscript as it stands now is very informative for readers.**

b. Lines 133: Fig 1. Map of Democratic Republic of Congo: the title of the map does not state what is being presented, i.e. administrative divisions (provinces)? How should the different colors be interpreted? There is no legend.

**Response: Thanks for the comments. This was fixed in the revised manuscript and reads now “Map of Democratic Republic of the Congo (26 provinces)”.**

c. Line 187: the residual variance is mentioned in line 193, but not in the equation appearing in line 187.

**Response: The authors thank the reviewer; however, the comment has already been addressed in the manuscript. Indeed, the equation** $Y_{i}\mathcal{\sim N}\left( \mu_{i},\sigma^{2} \right)$ **is clear about the Bayesian spatially varying coefficient (SVC) model. In this equation, the variance is included. The following equation is the estimation of the mean** $\mu_{i}$

d. Lines 236-239: there is a discrepancy between the text and Figure 2. The text states that the rural curve is slightly shifted to the left of the urban curve, suggesting higher levels of stunting in rural children compared with urban children. However, Table 1 shows a lower mean HAZ in urban children compared to rural children. Furthermore, the urban curve (blue) is shifted to the left compared to the rural curve (pink). The authors should harmonize the color code of the legend in Fig. 3 with the results presented in Table 1.

**Response: Thanks for the comments. We have incorporated them in the revised manuscript to make the text more comprehensible.**

e. Lines 279-280: The text states that additional details are provided in Table 2 and does not explain these details. The authors should provide an adequate interpretation of the data presented in Table 2.

**Response: The authors thank the reviewer. The manuscript provides now adequate interpretations of data presented in Table 2.**

3. Other points:
None

**Reviewer #2:**

Comment 1: In the context sub section of the method section, a global nutrition report is mentioned and it is reported that "The ~~145~~ Global Nutrition Report further underscores chronic vulnerabilities linked to poverty, inadequate ~~146~~ infrastructure, and prolonged instability [reference 24]." Does this reference has a specific connection with the study area description? If not, I believe this section shall be moved to the introduction section.

**Response: The authors thank the reviewer for the great comment; however, the reference points to the context because the country is still facing serious nutrition issues as many reports, including the one cited have shown. Therefore, pointing out to that reinforces the need of “nutrition” research in the setting.**

Comment 2: In the outcome variable subsection of the method section it is mentioned that "Dichotomizing HAZ discards information about the magnitude of deviation from the reference ~~157~~ standard. For example, a child with HAZ = −2.01 is treated identically to one with HAZ ~~=158~~ −4.5". According to the WHO HFA classification a Z score less than -2 and less than -3 are not the same. A Z score <-2 is described as stunting while a Z score of <-3 is as severe stunting. I believe, the description shall be narrated according to the WHO guideline.

**Response: Thank you for the comment. The manuscript clarified that and read now: “Most empirical studies define stunting using the WHO threshold of a height‑for‑age z‑score (HAZ) below –2, operationalized as a binary outcome rather than a continuous measure. While this dichotomous indicator facilitates comparability across studies, it compresses substantial variation in child growth outcomes. Treating HAZ as binary discards information about the severity of deviation from the reference standard—for instance, a child with HAZ = –2.01 is classified identically to one with HAZ = –4.5, yet both are considered fundamentally different from a child with HAZ = –1.99, despite nearly identical nutritional status. This loss of granularity reduces statistical power, obscures dose–response relationships, and masks gradients in chronic growth faltering. In this study, we therefore model height‑for‑age as a continuous outcome, preserving the full distribution of growth trajectories and enabling the detection of nonlinearities and heterogeneous effects.”**

Comment 3: In the result section, specifically Figure 3, the prevalence of stunting in the study area is reported from very low to very high, what is the cut off reference to categorize the prevalence as it is currently described in the result section? I believe, the cut off point for the prevalence with its standard references shall be reflected.

**Response: Thank you for the comment. To avoid the confusion, the map has been redesigned to keep only the percentage of stunted children under 5 years; and the text has been adjusted accordingly.**
