# Supplementary material for: Child malnutrition in the Democratic Republic of the Congo: A Bayesian Gaussian regression analysis using the 2023–24 demographic and health survey
Source: PLoS One. 2026 Oct 8;21(10):e0360056. doi: 10.1371/journal.pone.0360056 (PMC13648973; doi:10.1371/journal.pone.0360056)
Supplement: Supplementary file 2 [file pone.0360056.s002.docx]

**Manuscript Review report**

Manuscript title: Child Malnutrition in the Democratic Republic of the Congo: A Bayesian Gaussian Regression Analysis Using the 2023–24 Demographic and Health Survey

1. Summary of the research and overall impression

***Research question, claims, conclusions and context:*** The authors explored the geographical distribution of the prevalence, and correlates for child malnutrition in the Democratic Republic of the Congo (DRC) based on the Height for age (HAZ) indicator and by fitting nonlinear Bayesian-gaussian secondary regression analysis to data from DRC’s 2023–24 Demographic and Health Survey. They claim that the findings confirm the widespread prevalence of stunting in this vast country with important geographic inequalities. Thus, they conclude that their approach helps providing evidence for focusing interventions on most affected regions of the country and towards significant associated factors. This research reproduces the methods used elsewhere in Africa and other regions of the world confronted with child malnutrition, and provide similar conclusions.

***Strengths*** *and* ***weaknesses:*** The manuscript has the merit of using data from the Demographic and Health Survey 2023-2024 which is both a recent and a methodologically sound study covering the whole RDC. The analytical tools used permit to explore nonlinear associations using regression to control for potential confounders. The geographic mapping of stunting is very useful for focused interventions.

However, as the authors point out, the cross-sectional nature of the data does not permit to make causal inferences abouts the factors contributing to child stunting. Moreover, the analysis is only powered to the level of the province which represents the intermediate level in the health structure of the DRC. The true operational level, much more suited for interventions, is the health zone (health district) level. In addition, in relation to child chronic malnutrition, the distinction made in this paper among the key covariates and the potential confounders is unclear.

***Overall recommendation****:* The authors recommend to use spatial analytical tools to identify malnutrition high-risk regions and to inform targeted public health interventions. I agree with authors’ recommendation to use coordinated efforts to tackle both immediate needs and broader determinants of child health. I recommend that the authors take into account the minor issues raised below so that the review process of this manuscript proceed forward.

2. Evidence and examples

**Major Issues**

None

**Minor Issues**

a. Lines 1-3: Title: the authors should consider reformulating the title in order to clearly announce the content of the manuscript.

*The authors opted to keep the title unchanged. This is a minor issue.*

b. Lines 133: Fig 1. Map of Democratic Republic of Congo: the title of the map does not state what is being presented, i.e. administrative divisions (provinces)? How should the different colors be interpreted? There is no legend.

*The authors revised the title of the map, making it clearer to interpret.*

c. Line 187: the residual variance is mentioned in line 193, but not in the equation appearing in line 187.

*The equation reflects the residual variance, indeed.*

d. Lines 236-239: there is a discrepancy between the text and Figure 2. The text states that the rural curve is slightly shifted to the left of the urban curve, suggesting higher levels of stunting in rural children compared with urban children. However, Table 1 shows lower mean HAZ in urban children compared to rural children. Furthermore, the urban curve (blue) is shifted to the left compared to the rural curve (pink). The authors should harmonize the color code of the legend in Fig. 3 with the results presented in Table 1.

*The authors revised the text related to Figure 2 and harmonized the legend.*

e. Lines 279-280: The text states that additional details are provided in Table 2 and does not explain these details. The authors should provide adequate interpretation of the data presented in Table 2.

*The authors added the missing details.*

3. Other points:

None
