# Supplementary material for: Child malnutrition in the Democratic Republic of the Congo: A Bayesian Gaussian regression analysis using the 2023–24 demographic and health survey
Source: PLoS One. 2026 Oct 8;21(10):e0360056. doi: 10.1371/journal.pone.0360056 (PMC13648973; doi:10.1371/journal.pone.0360056)
Supplement: Supplementary file 3 [file pone.0360056.s003.docx]

**Manuscript Review report**

Manuscript title: Child Malnutrition in the Democratic Republic of the Congo: A Bayesian Gaussian Regression Analysis Using the 2023–24 Demographic and Health Survey

a. Lines 1-3: Title: the authors should consider reformulating the title in order to clearly announce the content of the manuscript.

*The authors opted to keep the title unchanged. This is a minor issue.*

**Response: The authors contend that the title is complete and comprehensive as it stands now. We appreciate the reviewer’s recommendation to keep it as it is now.**

b. Lines 133: Fig 1. Map of Democratic Republic of Congo: the title of the map does not state what is being presented, i.e. administrative divisions (provinces)? How should the different colors be interpreted? There is no legend.

*The authors revised the title of the map, making it clearer to interpret.*

**Response: The authors think that this comment was addressed in Revision #1 (R1 hereafter) and reads “Map of 26 provinces of the Democratic Republic of Congo”. In the Democratic Republic of Congo, the “province” is the first administrative level (26 provinces), followed by Territories (a total of 145 territories in the country). In the manuscript, the first administrative level (or 26 provinces) is mapped, and each province is named. For further details, see** [**https://en.wikipedia.org/wiki/Democratic_Republic_of_the_Congo#Government_and_politics**](https://en.wikipedia.org/wiki/Democratic_Republic_of_the_Congo#Government_and_politics)

c. Line 187: the residual variance is mentioned in line 193, but not in the equation appearing in line 187.

*The equation reflects the residual variance, indeed.*

**Response: The authors explained that in R1. Indeed, the equation** $\boldsymbol{Y}_{\boldsymbol{i}}\mathcal{\sim N}\left( \boldsymbol{\mu}_{\boldsymbol{i}}\mathbf{,}\boldsymbol{\sigma}^{\boldsymbol{2}} \right)$ **contains the residual variance** $\left( \boldsymbol{\sigma}^{\boldsymbol{2}} \right)$**. The equation in line 187 that the reviewer refers to only shows how** $\boldsymbol{\mu}_{\boldsymbol{i}}$ **is estimated. Therefore, the equation is complete and no change is required. To better clarify, it reads as follows “where:** $\boldsymbol{\beta}_{\boldsymbol{0}}\mathbf{,}\boldsymbol{\beta}_{\boldsymbol{1}}\mathbf{,}\boldsymbol{\beta}_{\boldsymbol{2}}\mathbf{,}\boldsymbol{\beta}_{\boldsymbol{3}}$ **are fixed effects;** $\boldsymbol{u}_{\boldsymbol{p}_{\boldsymbol{i}}}\mathcal{\sim N}\left( \boldsymbol{0}\mathbf{,}\boldsymbol{\sigma}_{\boldsymbol{u}}^{\boldsymbol{2}} \right)$ **is a province-level random effect;** $\boldsymbol{f}_{\text{spatial}}\left( \boldsymbol{s}_{\boldsymbol{i}} \right)$ **is a spatial intercept;** $\boldsymbol{f}_{\text{spatial, edu}}\left( \boldsymbol{s}_{\boldsymbol{i}} \right)$ **is a spatially varying coefficient for maternal education. In the equation,** $\boldsymbol{\sigma}^{\boldsymbol{2}}$ **is the residual variance of** $\boldsymbol{Y}_{\boldsymbol{i}}$ **conditional on** $\boldsymbol{\mu}_{\boldsymbol{i}}$ **(i.e., the unexplained variability remaining after accounting for the fixed effects, the province random effect, and the spatial terms).” See page 10**

d. Lines 236-239: there is a discrepancy between the text and Figure 2. The text states that the rural curve is slightly shifted to the left of the urban curve, suggesting higher levels of stunting in rural children compared with urban children. However, Table 1 shows lower mean HAZ in urban children compared to rural children. Furthermore, the urban curve (blue) is shifted to the left compared to the rural curve (pink). The authors should harmonize the color code of the legend in Fig. 3 with the results presented in Table 1.

*The authors revised the text related to Figure 2 and harmonized the legend.*

**Response: Thank you for this comment. We have replaced the original narrative with this “With respect to residence, the urban curve is shifted slightly to the left of the rural curve, indicating marginally higher levels of stunting among urban children compared with rural children. This pattern is consistent with the lower mean HAZ observed among urban children relative to rural children in Table 1.” See page 13.**

e. Lines 279-280: The text states that additional details are provided in Table 2 and does not explain these details. The authors should provide adequate interpretation of the data presented in Table 2.

*The authors added the missing details.*

**Response: Thank you for this comment. Table 2 is fully described as it reports “Posterior estimates of fixed, nonlinear, and spatial effects on height-for-age z-scores (HAZ)” see page 19, lines 303-315.**

3. Other points:

None
