# Supplementary material for: Child malnutrition in the Democratic Republic of the Congo: A Bayesian Gaussian regression analysis using the 2023–24 demographic and health survey
Source: PLoS One. 2026 Oct 8;21(10):e0360056. doi: 10.1371/journal.pone.0360056 (PMC13648973; doi:10.1371/journal.pone.0360056)
Supplement: Peer review history

# Peer review: Decision Letter 0

**DOI:** 10.1371/journal.pone.0360056.r001

**Authors:** Md. Moyazzem Hossain

9 Jun 2026

PLOS One

Dear Dr. Tsala Dimbuene,

Thank you for submitting your manuscript to PLOS ONE. After careful consideration, we feel that it has merit but does not fully meet PLOS ONE’s publication criteria as it currently stands. Therefore, we invite you to submit a revised version of the manuscript that addresses the points raised during the review process.

======================================

- In Fig 1, it is suggested that the scale, latitude, longitude, and north indication be added. Also, remove the title used in the map.
- In Fig 3, it is suggested that the scale, latitude, longitude, and north indication be added. Also, remove the title used in the map.
- In Fig 6, it is suggested that the scale, latitude, longitude, and north indication be added.
- Add strengths and limitations as a separate section.
- It is recommended to provide the reproducible code as a supplementary file.

======================================

Please submit your revised manuscript by Jul 24 2026 11:59PM. If you will need more time than this to complete your revisions, please reply to this message or contact the journal office at plosone@plos.org. When you're ready to submit your revision, log on to https://www.editorialmanager.com/pone/ and select the 'Submissions Needing Revision' folder to locate your manuscript file.

- A letter that responds to each point raised by the academic editor and reviewer(s). You should upload this letter as a separate file labeled 'Response to Reviewers'.
- A marked-up copy of your manuscript that highlights changes made to the original version. You should upload this as a separate file labeled 'Revised Manuscript with Track Changes'.
- An unmarked version of your revised paper without tracked changes. You should upload this as a separate file labeled 'Manuscript'.

If you would like to make changes to your financial disclosure, please include your updated statement in your cover letter. Guidelines for resubmitting your figure files are available below the reviewer comments at the end of this letter.

If applicable, we recommend that you deposit your laboratory protocols in protocols.io to enhance the reproducibility of your results. Protocols.io assigns your protocol its own identifier (DOI) so that it can be cited independently in the future. For instructions see: https://journals.plos.org/plosone/s/submission-guidelines#loc-laboratory-protocols. Additionally, PLOS ONE offers an option for publishing peer-reviewed Lab Protocol articles, which describe protocols hosted on protocols.io. Read more information on sharing protocols at https://plos.org/protocols?utm_medium=editorial-email&utm_source=authorletters&utm_campaign=protocols.

As the corresponding author, your ORCID iD is verified in the submission system and will appear in the published article. PLOS supports the use of ORCID, and we encourage all coauthors to register for an ORCID iD and use it as well. Please encourage your coauthors to verify their ORCID iD within the submission system before final acceptance, as unverified ORCID iDs will not appear in the published article. *Only* the individual author can complete the verification step; PLOS staff *cannot* verify ORCID iDs on behalf of authors.

We look forward to receiving your revised manuscript.

Kind regards,

Md. Moyazzem Hossain, PhD

Academic Editor

PLOS One

Journal Requirements:

When submitting your revision, we need you to address these additional requirements.

1. Please ensure that your manuscript meets PLOS ONE's style requirements, including those for file naming. The PLOS ONE style templates can be found at

https://journals.plos.org/plosone/s/file?id=wjVg/PLOSOne_formatting_sample_main_body.pdfand https://journals.plos.org/plosone/s/file?id=ba62/PLOSOne_formatting_sample_title_authors_affiliations.pdf

2. Please include your full ethics statement in the ‘Methods’ section of your manuscript file. In your statement, please include the full name of the IRB or ethics committee who approved or waived your study, as well as whether or not you obtained informed written or verbal consent. If consent was waived for your study, please include this information in your statement as well.

3. We note that Figure(s) 1, 3, 6 in your submission contain [map/satellite] images which may be copyrighted. All PLOS content is published under the Creative Commons Attribution License (CC BY 4.0), which means that the manuscript, images, and Supporting Information files will be freely available online, and any third party is permitted to access, download, copy, distribute, and use these materials in any way, even commercially, with proper attribution. For these reasons, we cannot publish previously copyrighted maps or satellite images created using proprietary data, such as Google software (Google Maps, Street View, and Earth). For more information, see our copyright guidelines: http://journals.plos.org/plosone/s/licenses-and-copyright.

We require you to either (1) present written permission from the copyright holder to publish these figures specifically under the CC BY 4.0 license, or (2) remove the figures from your submission:

a. You may seek permission from the original copyright holder of Figure(s) 1, 3, 6 to publish the content specifically under the CC BY 4.0 license.

We recommend that you contact the original copyright holder with the Content Permission Form (http://journals.plos.org/plosone/s/file?id=7c09/content-permission-form.pdf) and the following text:

“I request permission for the open-access journal PLOS ONE to publish XXX under the Creative Commons Attribution License (CCAL) CC BY 4.0 (http://creativecommons.org/licenses/by/4.0/). Please be aware that this license allows unrestricted use and distribution, even commercially, by third parties. Please reply and provide explicit written permission to publish XXX under a CC BY license and complete the attached form.”

Please upload the completed Content Permission Form or other proof of granted permissions as an "Other" file with your submission.

In the figure caption of the copyrighted figure, please include the following text: “Reprinted from [ref] under a CC BY license, with permission from [name of publisher], original copyright [original copyright year].”

b. If you are unable to obtain permission from the original copyright holder to publish these figures under the CC BY 4.0 license or if the copyright holder’s requirements are incompatible with the CC BY 4.0 license, please either i) remove the figure or ii) supply a replacement figure that complies with the CC BY 4.0 license. Please check copyright information on all replacement figures and update the figure caption with source information. If applicable, please specify in the figure caption text when a figure is similar but not identical to the original image and is therefore for illustrative purposes only.

The following resources for replacing copyrighted map figures may be helpful:

USGS National Map Viewer (public domain): http://viewer.nationalmap.gov/viewer/

The Gateway to Astronaut Photography of Earth (public domain): http://eol.jsc.nasa.gov/sseop/clickmap/

Maps at the CIA (public domain): https://www.cia.gov/library/publications/the-world-factbook/index.html and https://www.cia.gov/library/publications/cia-maps-publications/index.html

NASA Earth Observatory (public domain): http://earthobservatory.nasa.gov/

Landsat: http://landsat.visibleearth.nasa.gov/

USGS EROS (Earth Resources Observatory and Science (EROS) Center) (public domain): http://eros.usgs.gov/#

Natural Earth (public domain): http://www.naturalearthdata.com/

4. If the reviewer comments include a recommendation to cite specific previously published works, please review and evaluate these publications to determine whether they are relevant and should be cited. There is no requirement to cite these works unless the editor has indicated otherwise.

5. Please review your reference list to ensure that it is complete and correct. If you have cited papers that have been retracted, please include the rationale for doing so in the manuscript text, or remove these references and replace them with relevant current references. Any changes to the reference list should be mentioned in the rebuttal letter that accompanies your revised manuscript. If you need to cite a retracted article, indicate the article’s retracted status in the References list and also include a citation and full reference for the retraction notice.

[Note: HTML markup is below. Please do not edit.]

Reviewers' comments:

Reviewer's Responses to Questions

**Comments to the Author**

1. Is the manuscript technically sound, and do the data support the conclusions?

Reviewer #1: Yes

Reviewer #2: Yes

**********

2. Has the statistical analysis been performed appropriately and rigorously?

Reviewer #1: Yes

Reviewer #2: Yes

**********

3. Have the authors made all data underlying the findings in their manuscript fully available?

The PLOS Data policy

Reviewer #1: Yes

Reviewer #2: Yes

**********

4. Is the manuscript presented in an intelligible fashion and written in standard English?

Reviewer #1: Yes

Reviewer #2: Yes

**********

Reviewer #1: Manuscript Review report

Manuscript title: Child Malnutrition in the Democratic Republic of the Congo: A Bayesian Gaussian Regression Analysis Using the 2023–24 Demographic and Health Survey

1. Summary of the research and overall impression

Research question, claims, conclusions and context: The authors explored the geographical distribution of the prevalence, and correlates for child malnutrition in the Democratic Republic of the Congo (DRC) based on the Height for age (HAZ) indicator and by fitting nonlinear Bayesian-Gaussian secondary regression analysis to data from DRC’s 2023–24 Demographic and Health Survey. They claim that the findings confirm the widespread prevalence of stunting in this vast country with important geographic inequalities. Thus, they conclude that their approach helps providing evidence for focusing interventions on most affected regions of the country and towards significant associated factors. This research reproduces the methods used elsewhere in Africa and other regions of the world confronted with child malnutrition, and provide similar conclusions.

Strengths and weaknesses: The manuscript has the merit of using data from the Demographic and Health Survey 2023-2024 which is both a recent and a methodologically sound study covering the whole RDC. The analytical tools used permit to explore nonlinear associations using regression to control for potential confounders. The geographic mapping of stunting is very useful for focused interventions.

However, as the authors point out, the cross-sectional nature of the data does not permit making causal inferences about the factors contributing to child stunting. Moreover, the analysis is only powered to the level of the province which represents the intermediate level in the health structure of the DRC. The true operational level, much more suited for interventions, is the health zone (health district) level. In addition, in relation to child chronic malnutrition, the distinction made in this paper among the key covariates and the potential confounders is unclear.

Overall recommendation: The authors recommend to use spatial analytical tools to identify malnutrition high-risk regions and to inform targeted public health interventions. I agree with authors’ recommendation to use coordinated efforts to tackle both immediate needs and broader determinants of child health. I recommend that the authors take into account the minor issues raised below so that the review process of this manuscript proceed forward.

2. Evidence and examples

Major Issues

None

Minor Issues

a. Lines 1-3: Title: the authors should consider reformulating the title in order to clearly announce the content of the manuscript.

Example: Distribution of Child Malnutrition in the Democratic Republic of the Congo: A Bayesian Gaussian Regression Analysis Using the 2023–24 Demographic and Health Survey data.

b. Lines 133: Fig 1. Map of Democratic Republic of Congo: the title of the map does not state what is being presented, i.e. administrative divisions (provinces)? How should the different colors be interpreted? There is no legend.

c. Line 187: the residual variance is mentioned in line 193, but not in the equation appearing in line 187.

d. Lines 236-239: there is a discrepancy between the text and Figure 2. The text states that the rural curve is slightly shifted to the left of the urban curve, suggesting higher levels of stunting in rural children compared with urban children. However, Table 1 shows a lower mean HAZ in urban children compared to rural children. Furthermore, the urban curve (blue) is shifted to the left compared to the rural curve (pink). The authors should harmonize the color code of the legend in Fig. 3 with the results presented in Table 1.

e. Lines 279-280: The text states that additional details are provided in Table 2 and does not explain these details. The authors should provide an adequate interpretation of the data presented in Table 2.

3. Other points:

None

Reviewer #2: Comment 1: In the context sub section of the method section, a global nutrition report is mentioned and it is reported that "The 145 Global Nutrition Report further underscores chronic vulnerabilities linked to poverty, inadequate 146 infrastructure, and prolonged instability [reference 24]." Does this reference has a specific connection with the study area description? If not, I believe this section shall be moved to the introduction section.

Comment 2: In the outcome variable subsection of the method section it is mentioned that "Dichotomizing HAZ discards information about the magnitude of deviation from the reference 157 standard. For example, a child with HAZ = −2.01 is treated identically to one with HAZ =158 −4.5". According to the WHO HFA classification a Z score less than -2 and less than -3 are not the same. A Z score <-2 is described as stunting while a Z score of <-3 is as severe stunting. I believe, the description shall be narrated according to the WHO guideline.

Comment 3: In the result section, specifically Figure 3, the prevalence of stunting in the study area is reported from very low to very high, what is the cut off reference to categorize the prevalence as it is currently described in the result section? I believe, the cut off point for the prevalence with its standard references shall be reflected.

**********

what does this mean?). If published, this will include your full peer review and any attached files.

If you choose “no”, your identity will remain anonymous but your review may still be made public.

**Do you want your identity to be public for this peer review?** For information about this choice, including consent withdrawal, please see our Privacy Policy

Reviewer #1: No

Reviewer #2: **Yes:**  tadele tesfean abraha

**********

[NOTE: If reviewer comments were submitted as an attachment file, they will be attached to this email and accessible via the submission site. Please log into your account, locate the manuscript record, and check for the action link "View Attachments". If this link does not appear, there are no attachment files.]

To ensure your figures meet our technical requirements, please review our figure guidelines: https://journals.plos.org/plosone/s/figures

You may also use PLOS’s free figure tool, NAAS, to help you prepare publication quality figures: https://journals.plos.org/plosone/s/figures#loc-tools-for-figure-preparation.

NAAS will assess whether your figures meet our technical requirements by comparing each figure against our figure specifications.

# Author comment: Author response to Decision Letter 1

**DOI:** 10.1371/journal.pone.0360056.r002

24 Jul 2026

We have attached the fie containing Responses to reviewers. Thanks

Attachment Submitted filename: PONE-D-26-15920 Point-by-point responses.docx

# Peer review: Decision Letter 1

**DOI:** 10.1371/journal.pone.0360056.r003

**Authors:** Md. Moyazzem Hossain

29 Aug 2026

Dear Dr. Tsala Dimbuene,

Thank you for submitting your manuscript to PLOS One. After careful consideration, we feel that it has merit but does not fully meet PLOS One’s publication criteria as it currently stands. Therefore, we invite you to submit a revised version of the manuscript that addresses the points raised during the review process.

Please submit your revised manuscript by Oct 13 2026 11:59PM. If you will need more time than this to complete your revisions, please reply to this message or contact the journal office at plosone@plos.org. When you're ready to submit your revision, log on to https://www.editorialmanager.com/pone/ and select the 'Submissions Needing Revision' folder to locate your manuscript file.

- A letter that responds to each point raised by the academic editor and reviewer(s). You should upload this letter as a separate file labeled 'Response to Reviewers'.
- A marked-up copy of your manuscript that highlights changes made to the original version. You should upload this as a separate file labeled 'Revised Manuscript with Track Changes'.
- An unmarked version of your revised paper without tracked changes. You should upload this as a separate file labeled 'Manuscript'.

If you would like to make changes to your financial disclosure, please include your updated statement in your cover letter. Guidelines for resubmitting your figure files are available below the reviewer comments at the end of this letter.

If applicable, we recommend that you deposit your laboratory protocols in protocols.io to enhance the reproducibility of your results. Protocols.io assigns your protocol its own identifier (DOI) so that it can be cited independently in the future. For instructions see: https://journals.plos.org/plosone/s/submission-guidelines#loc-laboratory-protocols. Additionally, PLOS One offers an option for publishing peer-reviewed Lab Protocol articles, which describe protocols hosted on protocols.io. Read more information on sharing protocols at https://plos.org/protocols?utm_medium=editorial-email&utm_source=authorletters&utm_campaign=protocols.

As the corresponding author, your ORCID iD is verified in the submission system and will appear in the published article. PLOS supports the use of ORCID, and we encourage all coauthors to register for an ORCID iD and use it as well. Please encourage your coauthors to verify their ORCID iD within the submission system before final acceptance, as unverified ORCID iDs will not appear in the published article. *Only* the individual author can complete the verification step; PLOS staff *cannot* verify ORCID iDs on behalf of authors.

We look forward to receiving your revised manuscript.

Kind regards,

Md. Moyazzem Hossain, PhD

Academic Editor

PLOS One

Journal Requirements:

1.If the reviewer comments include a recommendation to cite specific previously published works, please review and evaluate these publications to determine whether they are relevant and should be cited. There is no requirement to cite these works unless the editor has indicated otherwise.

2. Please review your reference list to ensure that it is complete and correct. If you have cited papers that have been retracted, please include the rationale for doing so in the manuscript text, or remove these references and replace them with relevant current references. Any changes to the reference list should be mentioned in the rebuttal letter that accompanies your revised manuscript. If you need to cite a retracted article, indicate the article’s retracted status in the References list and also include a citation and full reference for the retraction notice.

Additional Editor Comments:

Thanks for your revision. However, minor comments need to be addressed.

[Note: HTML markup is below. Please do not edit.]

Reviewer's Responses to Questions

**Comments to the Author**

Reviewer #1: All comments have been addressed

**********

2. Is the manuscript technically sound, and do the data support the conclusions?

Reviewer #1: Yes

**********

3. Has the statistical analysis been performed appropriately and rigorously?

Reviewer #1: Yes

**********

4. Have the authors made all data underlying the findings in their manuscript fully available?

The PLOS Data policy

Reviewer #1: Yes

**********

5. Is the manuscript presented in an intelligible fashion and written in standard English?

Reviewer #1: Yes

**********

Reviewer #1: Major Issues

None

Minor Issues

a. Lines 1-3: Title: the authors should consider reformulating the title in order to clearly announce the content of the manuscript.

Comment: The authors opted to keep the title unchanged. This is a minor issue.

b. Lines 133: Fig 1. Map of Democratic Republic of Congo: the title of the map does not state what is being presented, i.e. administrative divisions (provinces)? How should the different colors be interpreted? There is no legend.

Comment: The authors revised the title of the map, making it clearer to interpret.

c. Line 187: the residual variance is mentioned in line 193, but not in the equation appearing in line 187.

Comment: The equation reflects the residual variance, indeed.

d. Lines 236-239: there is a discrepancy between the text and Figure 2. The text states that the rural curve is slightly shifted to the left of the urban curve, suggesting higher levels of stunting in rural children compared with urban children. However, Table 1 shows lower mean HAZ in urban children compared to rural children. Furthermore, the urban curve (blue) is shifted to the left compared to the rural curve (pink). The authors should harmonize the color code of the legend in Fig. 3 with the results presented in Table 1.

Comment: The authors revised the text related to Figure 2 and harmonized the legend.

e. Lines 279-280: The text states that additional details are provided in Table 2 and does not explain these details. The authors should provide adequate interpretation of the data presented in Table 2.

Comment: The authors added the missing details.

3. Other points:

None

**********

what does this mean?). If published, this will include your full peer review and any attached files.

If you choose “no”, your identity will remain anonymous but your review may still be made public.

**Do you want your identity to be public for this peer review?** For information about this choice, including consent withdrawal, please see our Privacy Policy

Reviewer #1: No

**********

[NOTE: If reviewer comments were submitted as an attachment file, they will be attached to this email and accessible via the submission site. Please log into your account, locate the manuscript record, and check for the action link "View Attachments". If this link does not appear, there are no attachment files.]

To ensure your figures meet our technical requirements, please review our figure guidelines: https://journals.plos.org/plosone/s/figures

You may also use PLOS’s free figure tool, NAAS, to help you prepare publication quality figures: https://journals.plos.org/plosone/s/figures#loc-tools-for-figure-preparation.

NAAS will assess whether your figures meet our technical requirements by comparing each figure against our figure specifications.

Attachment Submitted filename: Revised manuscript review report.docx

# Author comment: Author response to Decision Letter 2

**DOI:** 10.1371/journal.pone.0360056.r004

20 Sep 2026

See attached

Attachment Submitted filename: Point-by-point Responses R2 Sep, 2026.docx

# Editor report: Decision Letter 2

**DOI:** 10.1371/journal.pone.0360056.r005

**Authors:** Md. Moyazzem Hossain

22 Sep 2026

Child Malnutrition in the Democratic Republic of the Congo: A Bayesian Gaussian Regression Analysis Using the 2023–24 Demographic and Health Survey

PONE-D-26-15920R2

Dear Dr. Tsala Dimbuene,

We’re pleased to inform you that your manuscript has been judged scientifically suitable for publication and will be formally accepted for publication once it meets all outstanding technical requirements.

Within one week, you’ll receive an e-mail detailing the required amendments. When these have been addressed, you’ll receive a formal acceptance letter and your manuscript will be scheduled for publication.

An invoice will be generated when your article is formally accepted. Please note, if your institution has a publishing partnership with PLOS and your article meets the relevant criteria, all or part of your publication costs will be covered. Please make sure your user information is up-to-date by logging into Editorial Manager at Editorial Manager® and clicking the ‘Update My Information' link at the top of the page. For questions related to billing, please contact billing support.

If your institution or institutions have a press office, please notify them about your upcoming paper to help maximize its impact. If they’ll be preparing press materials, please inform our press team as soon as possible -- no later than 48 hours after receiving the formal acceptance. Your manuscript will remain under strict press embargo until 2 pm Eastern Time on the date of publication. For more information, please contact onepress@plos.org.

Kind regards,

Md. Moyazzem Hossain, PhD

Academic Editor

PLOS One

Additional Editor Comments (optional):

Reviewers' comments:

# Editor report: Acceptance letter

**DOI:** 10.1371/journal.pone.0360056.r006

**Authors:** Md. Moyazzem Hossain

PONE-D-26-15920R2

PLOS One

Dear Dr. Tsala Dimbuene,

I'm pleased to inform you that your manuscript has been deemed suitable for publication in PLOS One. Congratulations! Your manuscript is now being handed over to our production team.

At this stage, our production department will prepare your paper for publication. This includes ensuring the following:

* All references, tables, and figures are properly cited

* All relevant supporting information is included in the manuscript submission,

* There are no issues that prevent the paper from being properly typeset

You will receive further instructions from the production team, including instructions on how to review your proof when it is ready. Please keep in mind that we are working through a large volume of accepted articles, so please give us a few days to review your paper and let you know the next and final steps.

Lastly, if your institution or institutions have a press office, please let them know about your upcoming paper now to help maximize its impact. If they'll be preparing press materials, please inform our press team within the next 48 hours. Your manuscript will remain under strict press embargo until 2 pm Eastern Time on the date of publication. For more information, please contact onepress@plos.org.

You will receive an invoice from PLOS for your publication fee after your manuscript has reached the completed accept phase. If you receive an email requesting payment before acceptance or for any other service, this may be a phishing scheme. Learn how to identify phishing emails and protect your accounts at https://explore.plos.org/phishing.

If we can help with anything else, please email us at customercare@plos.org.

Thank you for submitting your work to PLOS One and supporting open access.

Kind regards,

PLOS One Editorial Office Staff

on behalf of

Professor Md. Moyazzem Hossain

Academic Editor

PLOS One
